# Evolution over the last 40 years of the assisted reproductive technologies in cattle - the Brazilian perspective for embryo transfer and resynchronization programs (part II)

**DOI:** 10.1590/1984-3143-AR2024-0058

**Published:** 2024-09-23

**Authors:** Guilherme Pugliesi, Isabella Rio Feltrin, Ana Clara Degan Mattos, Amanda Guimarães Silva, Karine Galhego Morelli, Thiago Kan Nishmura, José Nélio de Sousa Sales

**Affiliations:** 1 Universidade de São Paulo, Departamento de Reprodução Animal, Faculdade de Medicina Veterinária e Zooctecnia, Pirassununga, SP, Brasil; 2 Universidade Federal de Juiz de Fora, Departamento de Medicina Veterinária, Juiz de Fora, MG, Brasil

**Keywords:** doppler, ovulation, estrus, resynchronization, embryo transfer

## Abstract

The productivity of the beef and dairy industries depends directly on the reproductive efficiency and genetic gain of the herd, which are directly associated with the appropriate use of Assisted Reproductive Technologies (ARTs). The objective of this review is to show from a Brazilian perspective the evolution over the last 40 years of ARTs related to ovulation resynchronization programs and embryo transfer in cattle. Despite significant improvements and high fertility obtained in timed artificial insemination (TAI) protocols (Sales et al., 2024 - Part I), the improvement of the use of *in vitro-produced* embryos, development of resynchronization programs, and the advance in Doppler ultrasonography (Doppler-US) for reproductive assessments of bovine females were the ARTs that presented the greatest relevance on reproductive effectiveness in cattle. In the last seven years, the embryo transfer (ET) technology using *in vitro*-produced (IVP) embryos took over the conventional ET of *in vivo* produced embryos after donor’s superovulation. Also, procedures and pregnancy rates after ET of IVP embryos were improved in dairy and beef operations. The Doppler-US allows the identification of non-pregnant females at an early stage based on the evaluation of blood perfusion of the corpus luteum. Recent studies in beef and dairy cows indicate satisfactory accuracy when Doppler-US is used at 20-22 days after TAI. Consequently, super-early resynchronization programs have been developed and are being implemented in commercial programs, thereby facilitating earlier conception through the use of semen from superior bulls, providing genetic and economic improvements in herds. Likewise, the assessment of luteal function by Doppler-US allows the selection of embryo recipients with greater receptivity, and consequently may increase the effectiveness of timed ET programs.

## Introduction

Assisted reproductive technologies (ARTs) have positively impacted in dairy and beef industries over the last 40 years, leading to significant increases in productivity worldwide. Despite the great improvement in the use and protocols for timed-artificial insemination (TAI; discussed in Sales et al., 2024 - Part I), ARTs as resynchronization (Resynch) of ovulation and embryo transfer-associated technologies spread around the dairy and beef herds (Baruselli et al., 2019). Several of these ARTs were developed or were most used in Brazil mainly because Brazilian livestock plays a fundamental role in global meat production, and these ARTs have a great impact on reproductive efficacy in beef and dairy operations. Brazil has the largest commercial cattle herd in the world, which exceeds 187.5 million heads, and is the largest meat exporter (ABIEC, 2023). Despite these prominent positions, beef cattle farming in the country faces challenges related to low reproductive efficiency, compared with other influential nations in meat production, such as the United States (Baruselli et al., 2019). Given this scenario, improving productivity in this sector requires the constant evolution of livestock systems in all aspects of production, especially reproductive efficiency, aiming to achieve a greater number of pregnant cows in a short period post-calving.

The occurrence of postpartum anestrus and the prolonged interval between calving and a new conception in predominantly zebu females represent key factors associated with low reproductive rates in South America. Faced with this challenge, the development of several reproductive biotechniques appears as a promising strategy to improve reproductive efficiency, resulting in a significant increase in the pregnancy rate, a reduction in the interval between calving and conception, and the early identification of non-pregnant animals for a new opportunity to use TAI (Baruselli et al., 2017). In this scenario, the Resynch program allows a new TAI service in open females after the first service post-partum, eliminating the need for estrus detection, maximizing the use of selected bulls, and improving reproductive efficiency associated with genetic gain (Baruselli et al., 2017). Therefore, a significant increase in the use of Resynch in Brazil has been observed in TAI programs, especially in beef cattle.

In the diagnosis of pregnancy, early identification of non-pregnant females was developed using Doppler ultrasonography (Doppler-US), which enabled the early resynchronization (early-Resynch) of these non-pregnant females and reduced the calving-conception interval (Pugliesi et al., 2019a). In embryo-associated technologies, *in vitro* embryo production (IVP) increased drastically, which stimulated the greater transfer of *in vitro*-produced embryos than *in vivo* embryos and increased the reproductive rate of females with high-quality genetic material (Baruselli et al., 2019).

Therefore, we aimed with this review manuscript to present and discuss from a Brazilian perspective the evolution over the last 40 years of the main ARTs associated with resynchronization of ovulation and embryo transfer (ET) in cattle.

## Resynchronization of ovulation in dairy and beef cattle

An alternative for farms that have already adopted TAI as an approach to increase reproductive efficiency, especially when using sperm from bulls with high genetic merit, is the Resynch. This reproductive biotechnique involves the synchronization of ovulation in females previously subjected to TAI, which may occur before or after the conventional pregnancy diagnosis at 30 days post-TAI. The use of Resynch has increased due to the advantages of the TAI programs in reducing the calving interval, increasing pregnancy rates during the breeding season of beef cattle, and increasing the number of calves with greater genetic merit from AI (Baruselli et al., 2012; Pugliesi et al., 2019a).

For beef cattle, different Resynch programs can be used strategically within the breeding season and can be adapted to their reality, since the level of technology varies among Brazilian farms. Different from beef cattle operations that usually submit the females to reproduction in a specific breeding season of 90-120 days, for dairy cattle, the reproductive protocols usually will occur throughout the year and each specific routine usually occurs on specific days of the week. It is therefore essential to adapt reproductive management so that it would not interfere with other daily routines on the farm.

**Figure 1:**
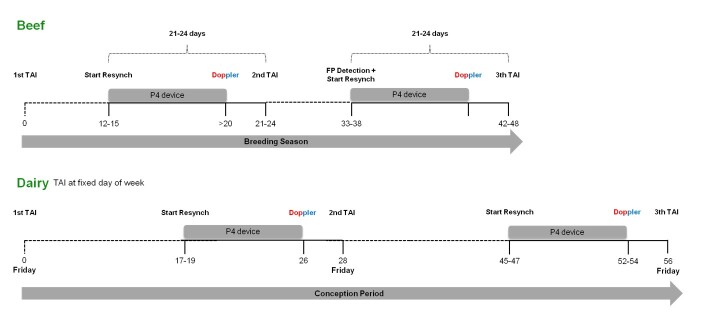
Schematic illustration of the super-early-Resynch strategy in TAI programs in beef and dairy cattle. In dairy, the TAI is usually performed on the same the day of week. Super-early-Resynch may start 12-15 days after TAI in beef and 17-19 days after TAI in dairy, resulting in an interval between TAIs of 21-24 days in beef and 28 days in dairy cattle. The Resynch associated with Doppler-US, enables 3 TAIs within 48 days in beef and 3 TAIs within 56 days in dairy herds. Abbreviations: TAI = timed-artificial insemination; Resynch = resynchronization; FP = false positive.

Based on the interval between the first service and the beginning of the protocol, Resynch programs can be classified as conventional, early, or super-early (Pugliesi et al., 2017). Conventional and early-Resynch programs are used more widely, mainly because they are associated with the use of B-mode ultrasound equipment. Conventional-Resynch is started at the time of the conventional pregnancy diagnosis (28 to 30 days post-TAI), and this program allows two TAIs within 40 days. In dairy cattle, because of the fixed days of the week for each step of the protocol, the conventional-Resynch usually begins 32 days after the first TAI, thus allowing a 42-day interval between TAI. However, to improve genetic and production gains, reproductive strategies must focus on improving the service rate and reducing the interval between services, without affecting ongoing pregnancies. In this regard, early-Resynch was developed, starting 22 days after TAI in all females, regardless of pregnancy diagnosis (Sá Filho et al., 2014; Pessoa et al., 2015). In early-Resynch, the pregnancy diagnosis is performed on the day the progesterone (P4) device is removed, and treatments with luteolytic drugs are only performed in non-pregnant animals at the time of P4 device removal. Thus, this protocol allows three TAIs within 64 days, achieving cumulative pregnancy rates of up to 87.8% (Crepaldi et al., 2017). In dairy cattle, considering the weekly approach, one strategy for implementing early-Resynch would be to bring the start of resynch forward by 7 days. Thus, starting 25 days after the first TAI is possible to achieve a 35-day interval between TAIs (Vasconcelos et al., 2014). In Brazil, conventional and early resynch protocols can be initiated for both dairy and beef cattle with the same recommended doses used in the synchronization protocols in the parity categories (discussed in Sales et al., 2024 - Part I). In super-early resynch, the use of estradiol benzoate (EB) associated with the P4 device is recommended, but it cannot exceeds the dose of 1 mg EB. The schematic model of the super-early-Resynch strategy in TAI programs in beef and dairy cattle is illustrated in Figure 1.

Estradiol is one of the triggers for the onset of luteolysis (Araujo et al., 2009; Pugliesi et al., 2011; 2012). Thus, in a Resynch protocol initiated without knowing the pregnancy status, an initial concern would be which drug and dose could be used without harming the ongoing pregnancy. Therefore, the use of EB or GnRH to induce a new follicular wave in early-Resynch was compared in beef and dairy cattle (Sá Filho et al., 2014; Vasconcelos et al., 2014). In beef females, it was observed that the pregnancy rate and pregnancy loss rate from the first TAI did not differ between females resynchronized with GnRH or 1mg EB. However, EB improved the pregnancy rate in Resynch. Because of the increased hepatic metabolization of steroid hormones in suckled or lactating cows (Sangsritavong et al., 2002), in a subsequent study, the pregnancy rate was compared between cows resynchronized with 1 or 2 mg of EB at 22 days post-TAI (Pessoa et al., 2015). In this study, the pregnancy rates from the first TAI did not differ between the groups; however, 2 mg of EB improved the pregnancy rate from Resynch. In dairy females, the same path was taken in the Resynch started at 25 days post-TAI (Vasconcelos et al., 2014). Firstly, it was observed the effectiveness of GnRH+P4 or BE+P4 to promote a new follicular wave. Following, another study (Vieira et al., 2015) was conducted to evaluate whether the administration of 2 mg EB 25 days post-TAI would influence ongoing pregnancy in Holstein cows. The pregnancy rate and pregnancy loss from the first TAI did not differ using GnRH, EB, or GnRH+EB. Therefore, early-Resynch protocols have been applied from day 22 after TAI using 2 mg EB in both beef and dairy cattle.

## Use of Doppler in cattle reproduction

Among the numerous technological advances in veterinary medicine over recent years, Doppler-US represents one of the most contemporary innovations. This technology detects variances between the frequencies of reflected waves and those emitted by the transducer, a phenomenon known as the "Doppler shift" (Szatmári et al., 2001). Within the context of blood circulation, these frequency differences stem from the movement of blood cells (Pugliesi et al., 2018). In cattle reproduction, Doppler ultrasound has been used for over two decades for the study of vascular perfusion in the ovaries, uterus, and vagina, as well as fetuses and placenta (reviewed by Herzog and Bollwein, 2007; Matsui and Miyamoto, 2009; Bollwein et al., 2016).

Based on the blood flow or perfusion, the Doppler-US provides important information about the functional status of a tissue or organ. The Doppler signal can be visualized on the ultrasound device screen in the form of spectral graphs or color-flow images. Spectral mode displays the Doppler signal as a time-velocity graph, illustrating fluctuations in blood flow throughout the cardiac cycle (Ginther, 2007). However, most studies on bovine reproduction, especially on those evaluating corpus luteum (CL) blood perfusion are performed based on the analysis of the colored area displayed in the color-flow Doppler image (Pugliesi et al., 2023). In color Doppler equipment, frequency differences are coded as colored signals over a conventional B-mode image (Ginther, 2007). The color flow mode generates real-time images of blood perfusion, providing easily interpretable visualizations of vascularization, such as CL (Pugliesi et al., 2023). The most direct way to quantify CL vascularization is through the evaluator's subjective scoring of the amount of colored area in the Doppler image (Siqueira et al., 2013; Guimarães et al., 2015). This method offers the advantage of encompassing the entire tissue scan and enables real-time diagnosis and swift decision-making, which are often essential in practical applications of the technology. Similar to other subjective scoring systems, this method relies on the evaluator's experience, with consistency typically improving over time (Pugliesi et al., 2023).

The use of Doppler-US in ARTs had 10 years ago a turning point which was the demonstration that Doppler imaging is a practical tool for the early diagnosis of non-pregnancy based on CL blood perfusion (Siqueira et al., 2013; Pugliesi et al., 2014). These pioneering studies in dairy and beef cattle demonstrated that early detection of non-pregnant cattle based on the CL blood perfusion starting from 20 days after TAI is highly accurate. Both studies use a subjective and real-time evaluation that is simpler and more practical than pixel counting to determine colored area and classify the CL as functional or not (Pugliesi et al., 2018). Siqueira et al. (2013) reported a high accuracy and almost 100% sensitivity on early diagnosis of non-pregnant dairy cattle (Holstein) using just CL blood perfusion as a criterion. In Nelore cattle, we observed nearly 100% sensitivity and 91% accuracy when considering luteal blood perfusion and size to determine pregnancy status on day 20 post-TAI (Pugliesi et al., 2014). Most importantly, Doppler-US is an accurate tool for early pregnancy diagnosis because there is a low possibility of erroneously diagnosing a pregnant cow as non-pregnant (false negatives near 0%) (Pugliesi et al., 2023).

Although there are well-known intrinsic differences in ovarian physiology between *B. indicus* and *B. taurus* females (Sartori et al., 2016), the accuracies of detecting non-pregnancy by Doppler on days 20 and 22 post-TAI in *Bos taurus* beef cattle (87% and 92%, respectively; Holton et al., 2022), were similar to those observed in *B. indicus* (Dalmaso de Melo et al., 2020). However, the proportion of false-positive results is increased and accuracy is reduced in *B. indicus* heifers compared with cows (Dalmaso de Melo et al., 2020). At least in Nelore animals, this difference is probably caused by a postponed spontaneous luteolysis in heifers than in suckled cows (Ataide et al., 2021).

## Early resynchronization of ovulation associated with Doppler imaging

The possibility of detecting pregnancy failures based on the identification of non-pregnant females that underwent luteolysis with high accuracy between 20 and 22 days after breeding opens a new possibility for ART. In beef cattle, this innovative approach enables super-early-Resynch, approximately 12 to 14 days after the first TAI or 5 to 7 days after timed ET (TET), followed by a new TAI or TET in open females at an interval of less than 24 days (Pugliesi et al., 2017, 2019a). This strategy reduces the interval between TAIs and TETs, significantly affecting the final pregnancy rate and the number of weaned calves, antecipating conception during the reproductive season (Baruselli et al., 2017). This strategy was pioneered by some research groups in Brazil and began to be used commercially by several companies and farms in South America. In dairy cattle, maintaining the weekly approach mentioned in previous topics, protocols have been proposed starting 17 or 19 days after the first TAI (Freitas et al., 2022; Neto et al., 2022). However, because of the greater proportion of false positive results in the diagnostic by Doppler-US compared with the gold standard by B-mode ultrasonography after day 28 of pregnancy (Siqueira et al., 2013; Ferraz et al., 2024), the use of the early-Resynch in dairy females is very modest.

The super-early-Resynch protocol typically begins close to maternal recognition of pregnancy, which relies on the adequate secretion of the interferon-τ glycoprotein by the conceptus (Spencer and Bazer, 2004). Thus, careful choice of drugs, doses, and timing is crucial in this protocol. Studies comparing the use of estradiol to resynchronize bovine females from the second post-breeding week onwards were performed based on the return to estrus as a method to evaluate the effect of treatments. However, the results varied according to parity order, dose, and time of treatment (Colazo et al., 2006; Machado et al., 2008). Consequently, new studies exploring the use of Doppler-US to assess CL functionality after estradiol treatment in the second week post-TAI have been initiated (Table 1).

**Table 1:** Studies evaluating the super-early resynchronization protocols associated to early detection of non-pregnant females by Doppler ultrasonography developed to reduce TAI interval in beef and dairy cattle.

**Authors**	**Type/Parity Order**	**n**	**Beginning of protocol (D0= first TAI)**	**Drug^1^, dose**	**FP rate^2^, %**	**Interval between TAIs, d**	**Conception rate at second TAI, %**
Vieira et al. (2015)	Dairy cows	90	D13	EB, 1.5 mg*	64	23	22.6
Penteado et al. (2016)	Beef cows	118	D14	SA-iP4, 100 mg	4.6	24	51
Colli et al. (2017)	Beef heifers	631	D14	SA-iP4, 50 mg	14.8	24	34.1
Santos et al. (2018)	Beef heifers		D14	No	-		35.7
		690		SA-iP4, 50 mg		24	37.0
				SA-iP4, 100 mg			39.1
Simões et al. (2018)	Beef heifers	1,065	D14	No	29	22-24	-
				SA-iP4, 50 mg			
Wallace et al. (2018)	Beef heifers	129	D14	SA-iP4, 50 mg	11.3	24	47.1
				LA-iP4 100 mg	18.9		12.1
Elliff et al. (2019)	Beef cows	151	D14	LA-iP4, 100 mg + EB, 2 mg	35	24	31.7
Morais et al. (2019)	Beef heifers	747	D14	SA-iP4 and LA-iP4, 50 mg	23.4ᴬ	22	40.6
			D15		13.3ᴮ	23	42.6
Pugliesi et al. (2019a)	Beef cows	376	D12	No	13.4	22	**44.6**ᴮ
				LA-iP4, 75 mg	17		**60.9**ᴬ
Andrade et al. (2020)	Beef heifers	300	D12	No	27.6	21	41.2
			D14	LA-iP4, 50mg	32.9	24	33.7
Cruz et al. (2020)	Beef heifers	1,390	D14	No	-	24	44.3
				EB, 1 mg			44.8
Motta et al. (2020)	Beef heifers	1,295	D14	No	19.5	24	**37.4ᴮ**
				EB, 1 mg	15.4		**47.0ᴬ**
				E_2_, 1 mg	15.2		**43.5ᴬᴮ**
Silva et al. (2021)	Beef heifers	1,116	D14	No	7.2	24	45.3
				EB, 1 mg	7.6		49.3
				SA-iP4, 140 mg	3.6		39.5
				SA-iP4, 140 mg+ EB, 1 mg	6.4		37.3
Vieira et al. (2021)	Beef heifers	677	D14	LA-iP4, 75 mg	11.3	24	**31.8ᴮ**
				EB, 1 mg	12		**45.9ᴬ**
Palhão et al. (2020)	Beef cows	1,002	D13	EB, 1 mg	9.3	23	42.8
Ataide et al. (2021)	Beef heifers and cows	1,258	D13	No	13	24	**38.2^B^**
				SA-iP4 ,100 mg			**43ᴬ**
Silva et al. (2022)	Beef cows	1,026	D14	EB, 1 mg	6ᴮ	24	47
				EB, 2 mg*	13ᴬ		42
Freitas et al. (2022)	Dairy cows	600	D19	No	29.0	28	32.2
				LA-iP4, 150 mg	37.9		30.2
				EB, 1 mg	32.3		37.3
Neto et al. (2022)	Dairy cows	300	D17	No	30ᴬ	28	32.4
				EB, 1 mg	18ᴮ		31

Legend: EB = estradiol benzoate; E_2_ = estradiol-17β; SA-iP4 = Short-acting progesterone; LA-iP4 = long-acting progesterone. ^1^ Drugs used associated to an intravaginal progesterone device. ^*^ Drugs not recommended to use associated to the progesterone device in super-early resynchronization protocols on 13 or 14 days after TAI because reduced the pregnancy rate at first TAI. ^AB^ Different superscripts within the same column of each study indicate a significant (P<0.05) difference between experimental treatments.

One of the first studies associating Doppler-US with Resynch protocols have been initiated on dairy cows. On day 13 after the first TAI, Vieira et al. (2014) allocated lactating Holstein cows into two experimental groups: Control group (which received only the P4-device) and the EB group (which received 1.5 mg EB + P4-device). In this study, it was observed that the group that received 1.5 mg of EB on day 13 post-TAI had a lower rate of active CL in the Doppler diagnosis and a lower pregnancy rate when compared with the group that only received the P4 device. Thus, the results of this study indicated that it was not safe to use 1.5 mg EB 13 days after the first TAI in dairy cows.

### Super-early resynchronization protocols for beef cattle

Due to the risks with the use of estradiol during the second week after insemination, in a first study conducted by our group (Motta et al., 2020), we aimed to evaluate the effect of estradiol use on the maintenance of pregnancy from first TAI, and the Resynch pregnancy rate. Thus, in the first experiment of this study (*Exp. 1*), 52 Nelore heifers received a P4-device and were divided into three experimental groups: Control group (no estradiol treatment), EB group (1 mg EB), and E2+P4 group (1 mg 17β-estradiol + 9 mg P4 i.m.). These heifers were daily evaluated using Doppler-US between days 14 and 22 after TAI. On day 22 post-TAI, devices were removed and Doppler diagnosis was performed. Heifers with active CL were considered pregnant and returned to confirmatory diagnosis on day 28 post-TAI. Regarding results, it was observed that both estradiol treatments anticipated luteolysis in non-pregnant females (18.9±0.5 days) when compared with the Control group (20.6±0.4 days). However, the estradiol did not impact the pregnancy rate of the first TAI, since the pregnancy rate did not differ among the groups. In a subsequent experiment using the same experimental design as *Exp.1*, a large number of animals was used (n=1,295). No difference was observed among the experimental groups for the proportion of animals with active CL on day 22 post-TAI (Control: 53.1%; EB: 53.1%; E2+P4: 50.4%), the pregnancy rate (Control: 43.3%; EB: 44.1%; E2+P4: 45.6%) and the potential pregnancy loss (Control: 19.5%; EB: 15.4%; E2+P4: 15.2%). Also, the Resynch pregnancy rate was greater in the EB group (47.0%) when compared with the Control group (37.4%). While the pregnancy rate of the E2+P4 group (43.5%) was intermediate.

Vieira et al. (2021), compared the use of 1 mg of EB or 75 mg of long-acting injectable P4, both associated with a P4-device, at the beginning of super-early-Resynch in Brangus and Braford heifers. Similar to the study by Motta et al. (2020), the authors reported that the pregnancy rate from the first TAI did not differ between the experimental groups. However, the pregnancy rate at the Resynch was greater in the group that received 1 mg EB when compared with the group that received injectable P4. Therefore, it was concluded that 1 mg EB is a safe and effective alternative for the super-early-Resynch of beef heifers.

Based on studies using estradiol for super-early-Resynch in beef heifers, studies were initiated in suckled beef cows. Therefore, Palhão et al. (2020), evaluated whether Resynch protocol using 1 mg of EB on day 13 after TAI could interfere with the pregnancy rate of the first TAI and the pregnancy of the Resynch. On day 13 post-TAI, 1,431 females were allocated into 2 experimental groups: Control (no treatment and returned to the management center only in the confirmation of pregnancy diagnosis at 30 days) and Resynch group (received 1 mg of EB + P4-device). On day 21 post-TAI, Doppler diagnosis was performed and the females without active CL were considered non-pregnant, following the protocol, and subjected to a second TAI two days later. The pregnancy rate at first TAI did not differ between groups (Control: 50.3%; Resynch group: 52.6%).

Although these promising results of super-early Resynch using 1mg EB, it has been suggested that in beef cows a greater dose of EB (2 mg) is required to induce an adequate synchronization of follicular emergence (Pessoa et al., 2015). Thus, our group tested whether increasing the EB dose in suckled beef cows would have an impact on ongoing pregnancy and the pregnancy rate of Resynch (Silva et al., 2022). In this study, on day 14 post-TAI, 1,026 Nelore cows received P4-device and were allocated into two experimental groups to receive 1 or 2 mg EB. Doppler diagnosis was performed eight days later. Cows with active CL were considered pregnant and had only devices removed. Cows without active CL were considered non-pregnant, followed the protocol, and were re-inseminated on day 24 post-TAI. The proportion of animals with active CL and the pregnancy rate from TAI was greater in the group treated with 1 mg EB (55.0% and 51.0%, respectively) when compared with the group treated with 2 mg EB (48.0% and 42.0%, respectively). In addition, potential pregnancy loss between days 22 and 35 was more than double in the 2 mg EB group compared with the group treated with 1 mg EB (13.0% *vs*. 6.0%). Resynch pregnancy rate did not differ between 1 or 2 mg EB groups, 47.0% and 42.0%, respectively. Finally, the cumulative pregnancy rate after two TAIs 24 days apart was greater in the EB 1 mg group (73%), when compared with the 2 mg EB group (64.0%). Together, these studies indicate that using EB at a dose of 1 mg associated with a P4-device is safe and efficient for super-early-Resynch in beef heifers and suckled beef cows.

Given the controversial results of the first studies using estradiol, the effects of the use of short-acting or long-acting injectable P4 (iP4) at different doses for super-early-resynch in heifers and cows were also evaluated. Rezende et al. (2016) applied 100 mg of short-acting iP4 on day 14 post-TAI in Nelore cows and observed that follicular emergence occurred 3.0±0.7 days after treatment. Thus, Penteado et al. (2016) compared the use of a P4-device associated with 100 mg of short-acting iP4 for super-early-Resynch at 14 days post-TAI with an early-Resynch starting at 22 days post-TAI in lactating Nelore cows. These authors observed no difference in pregnancy rate after first insemination (53.0% vs. 48.0%), as well as after Resynch (51.0% vs. 56.0%), and the cumulative pregnancy rate (75.0% [89/118] vs. 77.0% [97/126]) between super-early and early-Resynch systems, respectively. However, super-early Resynch improved service rate, every 21 days, from 66.0% to 87.5%.

Another study from our research group (Pugliesi et al., 2019a) indicated that the use of 75 mg of long-acting iP4 associated with a P4-device increased the pregnancy rate of lactating Nelore cows in the Resynch initiated at 12 days post-TAI compared with the group that received only one P4-device (60.9% [39/64] vs. 44.6% [25/56]). In this study, as the Doppler diagnosis was performed on day 20 post-TAI, the interval between the first and second TAI was only 22 days, and resulted in a 75% pregnancy rate with two TAIs. Likewise, Ataíde et al. (2021) evaluated the pregnancy rate of Nelore heifers (n=498) and cows (n=760) submitted to two consecutive Resynch’s, allowing for three TAIs in 48 days. In this study, 13 days after the first and second TAI, the females received a P4-device associated (P4-device + iP4) or not (only P4-device) with 100 mg of short-acting iP4. The Doppler-US was performed 22 days after the first and second TAIs. The pregnancy rate was greater in heifers and cows with P4-device + iP4 (38.0% [148/387] vs. 43.0% [178/411]). Thus, the use of P4 in its different presentations in super-early-resynch offers the potential to increase the pregnancy rate. More recently, another alternative of super-early-ressynch was proposed by Andrade et al. (2020) using only the P4 device at the beginning of the resynchronization. The protocol named Rebreed21 allows an interval between TAIs of 21 days resulting in satisfactory pregnancy rates in *B. indicus* beef females; however, the addition of EB treatments at the beginning of this protocol may further increase the effectiveness of this alternative, as EB has the potential to advance the luteolysis in non-pregnant females from first TAI resulting in reduction of false positive results at the Doppler-US diagnosis and improvement in dominant follicle growth for the second service.

Following the same line of reasoning, some studies have used super-early Resynch protocols in TET programs. Pellegrino et al. (2018) compared different strategies with P4 for early-Resynch of ovulation after ET in recipient beef cows. A total of 211 Nelore cows underwent a TET protocol and received an *in vitro*-produced embryo on D7 (D0=expected estrus). On day 13, all cows received a new P4 intravaginal device (CIDR, Zoetis, SP, Brazil) and were randomly divided into three groups: group 1 CIDR (no additional treatment), group 2 CIDR (an additional CIDR), and group CIDR+P4 (treatment with 100 mg of injectable P4 [Afisterone, Hertape Calier, SP, Brazil]). On day 22 , the devices were removed, and a Doppler diagnosis was performed. Heifers with active CL were considered to be pregnant and returned to confirmatory diagnosis on days 40 and 80 post-ovulation. The females without active CL were considered non-pregnant, following the protocol, and subjected to a second TET on day 31. Pregnancy rates on D22 were different (P<0.05) among treatment groups (43.8%^B^ [32/73] for 1CIDR; 57.5%^A^ [42/73] for 2CIDR; and 53.9%^AB^ [35/65] for 1CIDR+P4). However, pregnancy loss between D22 and D80 was greater (P<0.05) in the 2CIDR group (45.3%^A^ [19/42]) than in the 1CIDR (16.5%^B^ [5/32]), which consequently resulted in no difference (P>0.1) on pregnancy rates on D80 (37% [27/73] for 1CIDR; 31.5% [23/73] for 2CIDR; and 38.5% [25/65] for 1CIDR+P4). The utilization rate of cows at the second TET was greater (P<0.05) for 1CIDR (87.8% [36/41]) and 1CIDR+P4 (83.3% [25/30]) groups than 2CIDR group (71.0% [22/31]). Although the pregnancy rate after the second TET did not differ (P>0.1) among groups (48.7% [38/78]), the final pregnancy rate considering the first and second TET was different (63.0%^A^ [46/73] for 1CIDR; 43.8%^B^ [32/73] for 2CIDR; and 53.9%^AB^ [35/65] for 1CIDR+P4). Thus, the use of one CIDR without additional treatment with P4 at beginning of super-early Resynch associated with early detection of non-pregnant cows by Doppler results in desirable pregnancy and utilization rates and allows a 24d-interval between TETs in beef cattle. Based on this evolution, the use of super-early-Resynch increased the pregnancy rate and service rate, as it allows two TAIs or TETs 21 to 24 days apart, either using low-dose estradiol sources (up to 1 mg) or iP4 associated with the intravaginal P4 device at the beginning of Resync protocol.

### Super-early resynchronization protocols for dairy cattle

In dairy cattle, super-early-Resynch protocols took longer to develop when compared with beef cattle. One of the factors that contributed to a greater delay in the applicability of these protocols was the need to adapt the timing of the Doppler-US to the schedule of weekly visits by the veterinarian. Another factor considered was the greater rate of false positive results reported in dairy cattle (Siqueira et al., 2013; Ferraz et al., 2024).

Based on the results obtained in a previous study by our research group using beef heifers (Motta et al., 2021), a study was proposed to evaluate the performance of the Resynch protocol initiated 17 days after TAI, and the use of 1 mg of EB in this protocol in dairy cows (Neto et al., 2022). For this, 450 dairy cows were submitted to TAI (TAI=D0). On day 17 post-TAI, these cows were divided into 3 experimental groups (n=150 animals/group): Control (no Resynch), super-early-Resynch, and super-early-Resynch+EB. On day 31 post-TAI, non-pregnant cows of the Control group were submitted to the same hormonal protocol done in the first TAI and the second TAI on day 42 post-TAI. In the resynchronized groups, the Doppler-US was performed on day 24 post-TAI. On day 26 post-TAI, the devices were removed and the cows diagnosed as non-pregnant on Doppler received the second TAI on day 28 post-TAI. The pregnancy rate at first TAI did not differ significantly between the Control (44.0%), super-early-Resynch (47.0%), and super-early-Resynch+EB (47.0%) groups. The cumulative pregnancy rate within the 84 days of the experimental period was greater in cows submitted to super-early-Resynch (79.0%) than in the Control group (72.0%). Furthermore, the proportion of false positive rate between days 24 and 31 was lesser in the super-early-Resynch+EB group (18.0%) than in the super-early-Resynch group (30.0%). Thus, with this study, we concluded that the use of 1mg EB associated with a P4-device on day 17 post-TAI after TAI is preferable to increase the effectiveness of the super-early-Resynch protocol, as it is not harmful to the previous pregnancy, and reduces the proportion of false positive results by Doppler-US.

In addition, Freitas et al. (2022) evaluated the use of iP4 or EB to synchronize follicle waves in early-Resynch at 19 days after the first TAI on pregnancy rate and false positive rates in Holstein cows. Thus, in the *Exp. 1*, 600 lactating dairy cows were used. On day 19 after the first TAI cows were allocated into Control (without treatment) or iP4 (150 mg long-acting iP4) groups. On day 26 post-TAI, P4 devices were removed and Doppler-US was performed. On day 28 post-TAI, cows without active CL on Doppler-US were submitted to the second TAI. In *Exp. 2*, the same managements were performed, but on day 19 post-first TAI, cows were allocated into Control (without treatment) or EB (1 mg EB) groups. Therefore, treatment with P4i or EB in the early-Resynch protocol did not affect the pregnancy rate of the first TAI nor the pregnancy rate of the second TAI.

## Evolution of IVP

The history of bovine ET began in the late 1940s with the techniques for surgically recovering and transferring embryos (Umbaugh, 1949). These advancements culminated in the establishment of the modern embryo transfer (ET) industry in the early 1970s using surgical methods (Rowson and Dowling, 1949), and transitioned to non-surgical techniques at the end of the 70s (Drost et al., 1976). This change allowed the sector to expand rapidly, with Seidel (1981) reporting more than 17,000 pregnancies resulting from ET in North America by 1979. In the following decades, the development of IVP techniques (Brackett and Zuelke, 1993) has become increasingly efficient over time. Later, with the development of embryo freezing strategies (Hasler et al., 1995), there was a growing adoption of frozen and thawed IVP embryos around the world, although the proportion of fresh IVP embryos still remains greater.

Then, the year of 2017 there was a reversal in the production of bovine embryos, with the number of IVP exceeding those generated *in vivo* for the first time, indicating a significant change in preference for the *in vitro* technique (Viana et al., 2018). The popularity of IVP has grown not only in Brazil but also in other regions of the world, with the availability of sexed semen and improvements in technique efficiency driving its adoption, especially in dairy breeds (Pontes et al., 2010; Viana et al., 2017, 2018). This transition has resulted in a change in the profile of embryo production, with a greater proportion of embryos produced in taurine breeds or crosses, and in dairy breeds, reflecting a global trend (IBGE, 2017). Although the United States leads in total embryo numbers produced, Brazil remains a reference in IVP use, contributing significantly to global production and is in the first place for number of embryos transferred per year in the world (Viana, 2023). However, in 2020, a global pandemic affected several economic sectors, including embryo production. While the animal protein chain benefited from increased international demand, embryo production faced additional challenges due to the medium- and long-term return on investment (Viana, 2021).

After the pandemic, the Brazilian embryo market showed significant changes. A data survey carried out by Viana (2023) found a 3.9% increase in the absolute number of embryos reported to breeders’ associations. We could see a significant increase of 156.3% in the use of *in vivo* embryo collection, especially in the segment of taurine dairy breeds, such as Holstein. The IVP in the dairy segment fell slightly by 1.7%, while in the beef segment, there was an increase in both *in vivo* (+25.8%) and *in vitro* (+5.0%) production. Despite the variations, the dairy and beef segments' share of the total number of embryos reported remained relatively constant. The percentage of embryos produced *in vitro* and transferred after cryopreservation increased by 1.1%, corresponding to 35.1% of total transfers in 2022. In the case of embryos collected *in vivo*, for the first time since 2013, more transfers were carried out fresh than after cryopreservation (55.6% vs. 44.4%, respectively), reflecting the revival of this niche in 2022.

Despite the positive growth scenario for this biotechnology, we still need to consider some factors that will maintain this progress. One of the main limitation is the cost of preparing the recipients and to acquire the IVP embryo produced from high genetic merit donors and sires. Thus, enhancing extension services for producers, providing specialized services for preparing the recipients (estrous synchronization, and sanitary and nutritional managements) is crucial. Also, governamental or association programs may reduce the cost of the embryo. In this regard, Brazil has a program named SEBRAETEC-FIV founded by a non-profit private society (SEBRAE) in several states that allows the transfer of up to 40 embryos by farmer with a cost per confirmed pregnancy at less than 30% of the regular cost. In addition, training programs, continuing technological innovation, increased collaboration among universities, research institutes, industries, and producers, are essential to spread the IVP technology in small and medium-size farmers.

## Hormonal protocols for TET

The use of prostaglandin F2α (PGF2α) analogs for estrous synchronization in ET programs has been widely used in the past (Odde, 1990). However, the variability of the interval from administration of PGF2α to estrus and ovulation, as well as the low efficiency of estrus detection, leads to inefficiency on farms, where only 50% of treated recipients received an embryo 7 days after estrus (Bó et al., 2002). In the case of recipients with *Bos indicus* influence, this rate was less than 30% (reviewed in Bó et al., 2004). Therefore, to minimize these limitations, hormonal protocols that were initially developed for TAI are now being widely used in recipients submitted to TET programs (Bó et al., 2002, 2012). However, to enhance reproductive success, some premises must be aligned, such as the size of the ovulatory follicle, which has a positive association with the size of the CL and the circulation of P4 during diestrus (Senger, 2003). In addition, elevated P4 concentration during metestrus and early diestrus correlate with increased conceptus elongation (Mann and Lamming, 2001; O’Hara et al., 2014), thereby enhancing the probability of pregnancy following ET (McNeill et al., 2006; Meneghetti et al., 2009).

In view of this, several hormonal approaches have been employed to promote follicular growth and CL development, thereby enhancing pregnancy rate per TET (P/TET). Utilization of gonadotropins, such as equine chorionic gonadotropin (eCG) and human chorionic gonadotropin (hCG), are generally used to stimulate follicular and CL growth. Administering 200-400 IU eCG intramuscularly during the final growth of the dominant follicle (DF) is widely chosen in ovulation synchronization protocols for Zebu cows, as it improves pregnancy outcomes in anestrous cows and cycling heifers submitted to TAI programs (Baruselli et al., 2004; Pinto et al., 2020; Sales et al., 2011). Thus, eCG is used as a pre-ovulation strategy at the time of P4 device removal in TET protocols, promoting an increase in the dominant follicle size, circulating estradiol, and ovulation rate (Baruselli et al., 2004). Recently, a novel strategy carried out by our research group (Pugliesi et al., 2022) has been proposed in TAI programs using eCG at two moments to enhance the dominant follicle size and improve the pregnancy rates in suckled beef cows. In primiparous Nelore cows, dividing the eCG dose into two treatments (two days before and at P4 device removal) resulted in a 6.8% increase in pregnancy rates compared with a single dose at P4 device removal (Pugliesi et al., 2022). Supplementary to pre-ovulation gonadotrophic support, investigations have explored post-ovulatory strategies aimed at enhancing CL size on the day of embryo transfer. Thus, as hCG treatment has beneficial effects on luteogenesis by the similar LH activity, the CL size and P4 synthesis are increased in hCG-treated females at early diestrus (Dahlen et al., 2010; Maillo et al., 2014; Niswender et al., 2020).

In this context, in a recent study by our research group (Mattos et al., 2022), the impacts of different hCG doses on CL development and the effects of splitting the eCG dose in two moments during the ovulation synchronization protocol on P/TET were evaluated in primiparous crossbred recipients (Nelore X Angus; n = 727). In this study, splitting eCG dose during follicular growth or administering at least 1000 IU hCG at the onset of luteogenesis enhances CL development, improving P/TET in primiparous cows when these strategies are applied individually compared with females that received no additional treatment, but not when associated (control: 41%; splitted eCG: 53%; hCG: 52%; splitted eCG+hCG: 46%). The combination of strategies did not improve the pregnancy rate in this study possibly due to an imbalance between luteal cell proliferation and hypertrophy associated with eCG and hCG treatment (Prokopiou et al., 2014), as seen by the decrease in the CL blood perfusion at time of TET (Mattos et al., 2022). Splitting the eCG dose improved CL blood perfusion potentially through enhanced theca and granulosa cell development (Moura et al., 2015; Sousa et al., 2016), thereby modulating the uterus receptivity for embryo implantation.

Another strategy to improve the pregnancy per TET (P/TET) is use of iP4 prior to the TET protocols. In a recent study (Sales et al., 2024), the administration of 150mg of iP4 increased the P/TET [Control = 40.5% (159/393) *vs.* iP4 = 50.3 (188/374)] and the pregnancy rate [Control = 31.9% (159/498) vs. iP4 = 41.1 (188/458)] in recipients cows submitted to TET. However, the service rate was similar between the experimental groups [Control = 78.9% (393/498) vs. iP4 = 81.7 (374/458)]. These results suggest that the effects of iP4 prior to the synchronization of ovulation protocols may be related to uterine quality.

## Improvement of recipient selection in the TET programs

The maintenance of the recipient herd represents the largest economic expense in embryo transfer programs (Looney et al., 2006), so the best selection of the recipient is essential for the success of TET programs. In addition to the nutritional and sanitary management of the recipients, reproductive assessment at TET is essential and can be done through transrectal palpation and gray-scale ultrasonography to identify the CL; however, through these evaluations, we are not able to fully affirm the functionality of the luteal structure. Therefore, choosing recipients based exclusively on the size of the CL can result in the embryo being transferred into a female with an inadequate uterine environment (Pinaffi et al., 2015). Thus, these authors carried out the first study evaluating CL blood perfusion at TET in female recipients, which classified CL into high (>40%) or low (<40%) blood perfusion, and observed a greater pregnancy rate in high-perfusion females (48% vs. 0%). Consistent with the previous study, CL blood perfusion at the time of embryo transfer was higher in pregnant Holstein recipients than in non-pregnant (Kanazawa et al., 2016). However, the determination of luteal blood perfusion was based on the area of color signals indicating blood perfusion and the time-averaged maximum velocity at the base of the spiral artery, consuming a significant amount of time and having low applicability in large-scale programs.

**Figure 2:**
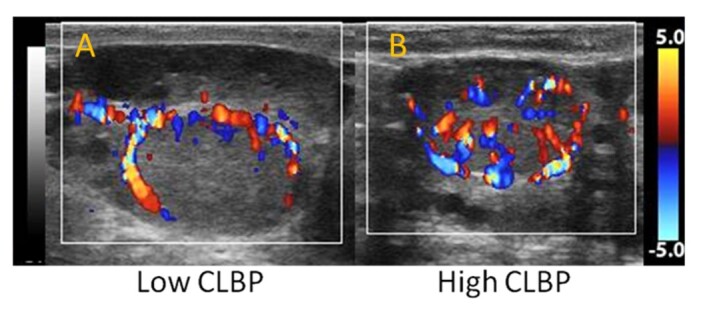
Color Doppler ultrasonographic images representing corpus luteum (CL) of bovine females with (A) low and (B) high blood perfusion (BP).

On this matter, in a study by our research group, we aimed to evaluate the impact of luteal characteristics at TET on the pregnancy rate in beef recipients, using real-time assessment of CL blood perfusion (Pugliesi et al., 2019b). In this study, we used two real-time techniques to subjectively assess luteal blood perfusion (i.e., the estimated proportion of colored signals on a scale of 0-100% and a scoring system [Figure 2]). In this retrospective study, 444 beef recipients were separated into three subgroups according to the luteal area and CL BP (low [<40%], medium [45-50%], or high [>55%)]). The results demonstrated that luteal blood perfusion assessed by Doppler-US evaluated at TET was the factor that had the greatest impact on the pregnancy outcome, showing a positive and linear result in beef recipients. Subsequently, new studies were carried out also evaluating luteal blood perfusion in real-time (Aragon et al., 2018; Munhoz et al., 2018; Silva et al., 2018). Despite not being significant, the pregnancy result per TET in recipients with high blood perfusion was about 1.3 to 2.3 times higher than those with lower CL BP in Holstein cows (42 vs. 18%; Aragon et al., 2018), Holstein-Gir heifers and cows (51.8 vs. 33%; Munhoz et al., 2018) and Angus heifers (60.6 vs. 48.1%; Silva et al., 2018). When this technique was performed in buffalo recipients, Silva (2020) observe a 2.5-fold increase in pregnancy in females with high CL BP (>50%) compared with those with low perfusion (<50%) at the TET.

In line with the results obtained in large ruminants, our group (Morelli et al., 2023) recently detected in horses that luteal blood perfusion was the only luteal sonography characteristic that affected pregnancy after *in vivo* ET, with recipient mares with high blood perfusion achieving greater pregnancy than those females with low blood perfusion (88.4% [38/43] vs. 65.5% [38/58]). Therefore, in large animal species, all these results indicate that luteal blood perfusion is strongly associated with pregnancy maintenance and can be the major factor in the selection of recipients with high receptivity.

Despite the positive results of luteal blood perfusion on pregnancy of embryo recipients, the mechanisms involved in the enhanced establishment of pregnancy have not been fully elucidated. The presumption that high luteal blood perfusion is associated with high P4 concentration during embryonic development was not supported, since the correlation with circulating P4 is weak for BP and moderate to high for the luteal area at the beginning of diestrus cattle or horses (Rocha et al., 2019; Morelli et al., 2023). Possibly, this high luteal blood perfusion may be associated with increased vascularization of the reproductive tract or resistance of the CL to regress. However, these alternatives still need to be investigated to obtain a better understanding of the CL blood perfusion mechanisms involved in pregnancy in cattle.

## Conclusion

Over the last 40 years, TAI has been the main ART utilized, enabling the expansion of AI on dairy and beef farms in Brazil, increasing service rates and genetic progress. This resulted in direct economic gains due to a positive impact on numer of calves borned and weigh at weaning in beef operations and reduction in the calving interval and increase in annual milk yeld in dairy systems (Baruselli et al., 2019). Understanding the potential of Doppler-US to detect females that failed to become pregnant after first service has allowed the development of a new strategy for super-early-Resynch. Through this reproductive biotechnique, it becomes possible to perform three TAIs in 48 days, resulting in a considerable increase in the service rate, a fact that leads to an earlier conception of the animals and a greater number of “early-born calves” in the calving season. Doppler-US is also a practical tool that has made it possible to improve reproductive efficiency in TET programs. Such advances have allowed better results in pregnancy rates, as well as the anticipation of conception within the breeding season for beef cattle. However, it should be noted that to obtain these advances, training and understanding of this imaging equipment is necessary, as it must be properly regulated, and the technician must be very well prepared to carry out the assessments correctly. Furthermore, the cost of the equipment must be taken into account, which is about 2 to 3 times more expensive than traditional devices. Regarding Resynch protocols, despite their consistent results, their commercial application must be very well studied, as they require much greater strategic planning than traditional protocols, both in terms of costs and concentrated labor. Therefore, the use of these biotechniques must be carefully analyzed for each property and often within groups of animals, as with proper planning these biotechniques can generate high reproductive and economic gains.
